# Detection and Localization of Tip-Burn on Large Lettuce Canopies

**DOI:** 10.3389/fpls.2022.874035

**Published:** 2022-05-12

**Authors:** Benjamin Franchetti, Fiora Pirri

**Affiliations:** ^1^Agricola Moderna, Milan, Italy; ^2^Alcor Lab, DIAG, Sapienza University of Rome, Rome, Italy; ^3^Deep Plants, Rome, Italy

**Keywords:** tip-burn detection and localization, self supervised segmentation, plant disease classification, segmentation of large canopies, indoor farming

## Abstract

Recent years have seen an increased effort in the detection of plant stresses and diseases using non-invasive sensors and deep learning methods. Nonetheless, no studies have been made on dense plant canopies, due to the difficulty in automatically zooming into each plant, especially in outdoor conditions. Zooming in and zooming out is necessary to focus on the plant stress and to precisely localize the stress within the canopy, for further analysis and intervention. This work concentrates on tip-burn, which is a plant stress affecting lettuce grown in controlled environmental conditions, such as in plant factories. We present a new method for tip-burn stress detection and localization, combining both classification and self-supervised segmentation to detect, localize, and closely segment the stressed regions. Starting with images of a dense canopy collecting about 1,000 plants, the proposed method is able to zoom into the tip-burn region of a single plant, covering less than 1/10th of the plant itself. The method is crucial for solving the manual phenotyping that is required in plant factories. The precise localization of the stress within the plant, of the plant within the tray, and of the tray within the table canopy allows to automatically deliver statistics and causal annotations. We have tested our method on different data sets, which do not provide any ground truth segmentation mask, neither for the leaves nor for the stresses; therefore, the results on the self-supervised segmentation is even more impressive. Results show that the accuracy for both classification and self supervised segmentation is new and efficacious. Finally, the data set used for training test and validation is currently available on demand.

## 1. Introduction

Plant stress detection is a long-standing research field and, among the stresses, tip-burn affecting, particularly, lettuce has been intensively studied, refer for example Termohlen and Hoeven (1965), Lutman (1919), Cox et al. (1976), and Gozzovelli et al. (2021).

Nowadays, the combination of new methods arising from computer vision and deep learning, the availability of new low-cost sensors together with increased attention on the transparency, quality, and healthiness of the farm to fork process is making plant stress analysis a challenging research topic.

**Figure 1:**
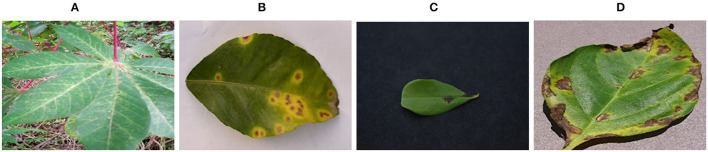
Images from the plant disease classification datasets: Cassava (Mwebaze et al., 2019), Citrus leaves (Rauf et al., 2019), PlantLeaves (Chouhan et al., 2019), and PlantVillage (Hughes and Salathe, 2016). The images clearly illustrate the difference with the proposed task of stress detection on large canopies. **(A)** Cassava, **(B)** Citrus leaves, **(C)** PlantLeaves, and **(D)** PlantVillage.

Classification of plant diseases is becoming a relevant topic thanks to a number of new data sets, such as PlantLeaves (Chouhan et al., 2019), PlantsDoc (Singh et al., 2020), PlantsVillage (Hughes and Salathe, 2016), Plantae-K (Vippon Preet Kour, 2019), Cassava (Mwebaze et al., 2019), Citrus leaves (Rauf et al., 2019), etc. made available as tensorflow datasets at *tensorflow.org*. Examples from these datasets are shown in Figure 1. These new datasets and their ease of accessibility have thrived the research improving deep learning models for stress detection applications.

**Figure 2:**
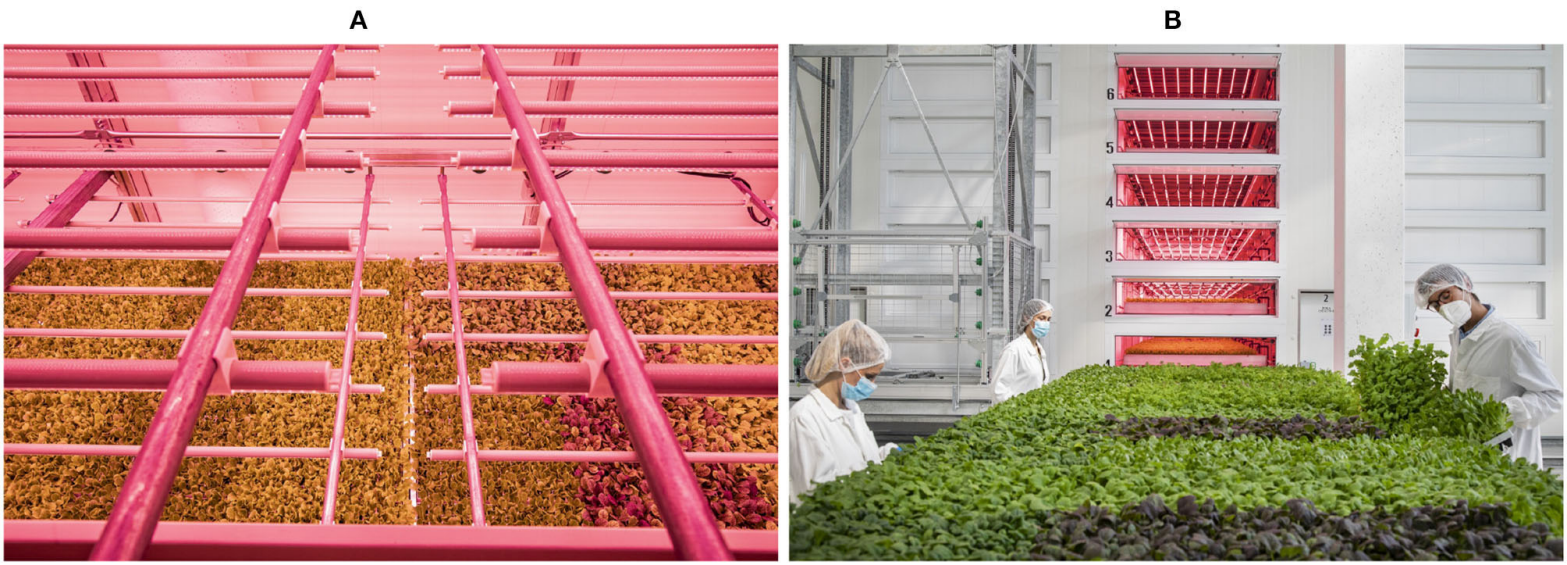
Large canopies of plants grown in Plant Factories **(A)**. In **(B)** we see the operators controlling the canopy to visually detect tip-burn, on the rolling tables.

A limit of the currently available datasets is their inadequateness for stress analysis in Controlled Environment Agriculture (CEA) and specifically in plant factories, where plants are grown indoors under artificial lights, densely packed together, and stacked on multiple layers. In such a highly densely growing conditions, the plants are compacted on tables of trays, and stress problems need to be studied from this specific perspective, as shown in Figure 2.

The detection and localization of stress in plant factories have to deal with complex surfaces agglomerating several plants, where the single leaf shape is not specifically relevant, and at the same time, stresses, such as tip-burn, occur on the leaf tip. Moreover, typically plants affected by tip-burn are few, sparse, and hidden in the canopy of other healthy leaves. The underlying cause of tip-burn is a lack of calcium intake by the plants. This, however, is a result of multiple factors, such as lack of airflow, high humidity, excessive lighting, inadequate watering, and nutrient supply. A key advantage of growing plants indoors is the possibility to control all aspects of the plant growth including the light recipe and climate, thereby providing the optimal mix of conditions to optimize plant development and quality. However, high-density crop production, limited dimensions, lack of natural ventilation, and the need for artificial lighting for photosynthesis makes plants grown in plant factories, especially, vulnerable to tip-burn. Consequently, tip-burn has become a metric for the healthiness of the plants, and being able to monitor its advent is extremely relevant in indoor growing conditions. By automatically detecting tip-burn, the vertical farm control software can adjust the growing recipes in real time to provide the plants with the optimal growing conditions.

In this work, we propose a novel model for tip-burn detection in lettuce that fills the gap between already explored techniques of deep learning applied to plant stress detection and their practical implementation in plant factories. Our work includes the realization of an adequate dataset made of real and generated images. Yet, to emphasize the generality of our contribution we have also tested our model on PlantLeaves (Chouhan et al., 2019), PlantsVillage (Hughes and Salathe, 2016), and Citrus leaves (Rauf et al., 2019) and compared with other works, whose results have a state of the art.

## 2. Related Works on Disease Detection

Plants disease detection is nowadays a quite impressive research field collecting methods and studies on a good diversity of diseases, crops, plant species, conditions, and contexts. In particular, most of the recent studies are based on deep learning methods, yet consider different cameras and datasets.

**Disease detection**. A number of approaches are based on dedicated sensors, such as hyperspectral cameras, or generate their own datasets. For example, Nagasubramanian et al. (2018) studied charcoal rot disease identification in soybean leaves by implementing a 3D Deep-CNN on data collected by a hyperspectral camera. Zhang et al. (2019) carried out a similar study using high-resolution hyperspectral images to detect the presence of yellow rust in winter wheat. Refer to Terentev et al. (2022) for a recent overview of hyperspectral approaches.

On the other hand, the publicly available datasets designed for disease classification, such as those introduced above, have played a crucial role in most of the deep learning methods.

Approaches exploiting the publicly available datasets have obtained very high accuracy for classification. For example, Agarwal et al. (2020) trained a CNN on tomato leaves images taken from the PlantVillage dataset obtaining 91.20% accuracy on 10 classes of diseases. On the other hand, on the same set of tomato classes, Abbas et al. (2021) obtained 97.11% accuracy with DenseNet121 + Synthetic images.

Patidar et al. (2020) obtained 95.38% accuracy in diseases classification on the Rice Leaf Disease Dataset (Prajapati et al., 2017) from the UCI Machine Learning Repository. Mishra et al. (2020) achieved 88.46% accuracy on corn plant disease detection, at the same time, obtaining real-time performance of a deep model capable of running on smart devices. Saleem et al. (2020) experimented a number of deep networks on the Plant Village dataset, proposing a comparative evaluation study between multiple CNNs and optimizers for the task of plant disease classification, in order to find the combination with the best performances, obtaining quite challenging results. Sharma et al. (2020) obtained 98.6% accuracy on PlantVillage by manually segmenting a subset of the images. Hassan and Maji (2022) obtain significant results on three datasets: 99.39% on PlantVillage, 99.66% on Rice, and 76.59% on imbalance cassava. Syed-Ab-Rahman et al. (2022) obtained 94.37% accuracy in detection and an average precision of 95.8% on the Citrus leaves dataset, distinguishing between three different citrus diseases, namely citrus black spot, citrus bacterial canker, and Huanglongbing.

Overall, results on the publicly available datasets are saturating toward super human performance, showing that new steps for diseases detection need to be taken.

Other digital images based deep learning approaches have experimented with their own datasets. Examples are DeChant et al. (2017) and Shrivastava et al. (2019). DeChant et al. (2017) consider the classification of the Northern Leaf Blight in maize plants, taking images of leaves in the field. While (Shrivastava et al., 2019) studied the strength of transfer learning for the identification of three different rice plant diseases. A recent review on computer vision and machine learning methods for disease detection is done in Barbedo (2019a), Abade et al. (2020), and Lu et al. (2021).

**Large canopies and tip-burn studies**. Tip-burn studies date back long ago (Lutman, 1919; Termohlen and Hoeven, 1965; Cox and McKee, 1976), essentially exploring causes induced by lack of nutrients absorption, such as in Son and Takakura (1989) and Watchareeruetai et al. (2018). As far as we know, only (Shimamura et al., 2019) conducted tip-burn identification in plant factories using GoogLeNet, for binary classification of single lettuce images. They check from manually collected images of a single plant whether it has tip-burn or not.

Similarly, in Gozzovelli et al. (2021), a dataset for tip-burn detection on large dense canopies of indoor grown plants is generated with specific attention to cover the data imbalance. To cope with the imbalance, a huge amount of data were generated with Wasserstein Generative Adversarial Network (GANs) and verified using the realism score of Kynkäänniemi et al. (2019). Classification was performed with two class-classifier architecture highly inspired from DarkNet-19, YOLOv2 backbone (Redmon and Farhadi, 2016), while the tip-burn region was identified preparing a ground-truth with a conditional random field, further generalized with a U-Net (Noh et al., 2015).

GANs were already used in Giuffrida et al. (2017) to generate Arabidopsis leaf using the number of leaves as the label. Similar to Gozzovelli et al. (2021) in Douarre et al. (2019), the authors explore segmentation at the canopy level, of apple scab. They augment the segmentation training set with conditional GANS.

**Plants stress and disease segmentation**. Segmentation for enhancing plant stress and disease detection has been explored by the works of Tian and LI (2004) and Zhang and Wang (2007). Most of the methods, even recently, tend to use image processing methods, such as filtering, thresholding, Gaussian mixtures, and color transforms to segment the disease or part of the leaf. Barbedo (2017) noted that when the disease symptoms show a difference in color with respect to surrounding areas, then ROI segmentation can be easily exploited. This observation has led to the study of the improvements in disease classification led by segmentation. This indeed was the choice in Gozzovelli et al. (2021) and Sharma et al. (2020), despite in the latter, segmentation is done manually. A leaf segmented version of Plant Village is used by Abdu et al. (2018) to introduce an automatic extended region of interest (EROI) algorithm for simplified detection. The segmentation of the disease is obtained by thresholding while the leaf segmentation is not treated and segmented leaf images are provided as a dataset. Following the work of Abdu et al. (2018) in Abdu et al. (2019), an extended EROI version is provided to study individual diseased segments, still based on a segmented version of PlantVillage, provided as a dataset.

In Douarre et al. (2019), the authors segment a canopy apple leaves extending the manual training set with cGAN (Mirza and Osindero, 2014) generated images. Sodjinou et al. (2021) propose a segmentation method to separate plants and weeds, based on initial semi-manual preprocessing, using cropping and thresholding, further U-Net semantic segmentation refines the segmentation and, finally, the results are post processed with a subtractive clustering algorithm.

As a matter of fact, despite the observation of Barbedo (2017), better and more generalized results can be obtained using deep learning methods that do not rely on specific image processing practices to come out with a segmentation result, as shown in other application fields.

**Weakly Self Supervised segmentation**. As far as we know, no method has so far explored self-supervised segmentation of plants disease, based on the class annotation only. Our work is the first one providing both the leaf segmentation (for PlantLeaves and PlantVillage, and Citrus Leaves) and the tip-burn stress segmentation without any manual annotation of pixel labels for segmentation.

We recall that weakly self-supervised segmentation (WSSS) is self-supervised segmentation using only image-level annotation. This means that only the information of the category in the image (e.g., “diseased” or “healthy”) is used to segment the object(s) of interest. Namely, the method consists of predicting a pseudo-label mask of the objects belonging to the class of interest, only relying on the image class label. Recent research has dedicated significant attention to the problem, introducing new methods based on weakly supervised learning, such as self training (Zou et al., 2018; Gu et al., 2020; Wang et al., 2020), domain adaptation (Pan et al., 2020; Yang and Soatto, 2020), noisy label learning (Xie and Huang, 2021) and class activation maps (CAM). CAM, introduced by Zeiler and Fergus (2014) and Zhou et al. (2016) localize the object of interest only relying on the image classes and backpropagating the probability to layers before the logits. The CAM-based methods have motivated a huge amount of works, such as Sun et al. (2020), Chan et al. (2021), Araslanov and Roth (2020), Yao and Gong (2020), and Wang et al. (2020). The method we propose in this work is WSSS using only the image class label, to segment the plants' lesions. The only available knowledge is whether the image represents a stressed or not-stressed region. Our method works on domains where the task is to generate pseudo label masks of quite small high-deformable shapes. Despite our elective application domain being large canopies of plants grown in plant factories, it can be used for other applications, as we show applying our method to publicly available datasets.

## 3. Materials and Methods

### 3.1. Data Collection

Since tip-burn manifests on the leaves tip, it is mandatory to acquire images with a top view of the whole table. We do so by taking images with an HR digital camera fixed above the rolling table shown in Figure 2. A table is a base on which plants are grown. Each table assembles into multiple trays, which in turn are further divided into multiple cells where plant seeds are placed.

We collected images of size 4.64E + 3×6.96E + 3×3 of the tables, using a camera Canon32.5 APS-C of 32.5 megapixels located above the rolling tables (shown in Figure 2). The whole set is made of 43 images, 30 for training and 13 for validation and testing. Images were collected in a period of tip-burn spread. As a tip burn is about 5×5 pixels in the camera image, we have devised a splitting process that allows to zoom into the table image. We split the 43 images of size 4, 640×6, 960×3 into smaller images of size 64×64×3, with an interface we have prepared for the task, and collected 2,127 images of tip-burn. We have automatically selected the same number of images of healthy plants, ensuring to be healthy by correlated with the stressed images from the same table. The images collected by splitting the original table images have been then augmented to finally obtain a training set of 16,323 images of tip-burn stressed and healthy plants, a validation set of 5,596 images and a test set of 1,399 images.

For the purpose of illustrating our method on other datasets, we used the PlantVillage, PlantLeaves, and CitrusLeaves datasets available on Tensorflow.org.

### 3.2. Method

**Figure 3:**
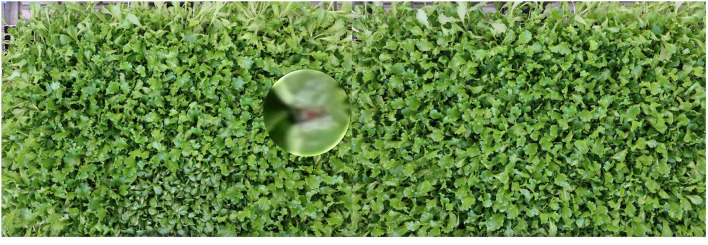
The problem: given the image of a large canopy find all regions with tip-burn. Because tip-burn regions are very small and maybe each other close, segmentation is better than simple localization with bounding boxes. We propose a novel method for weakly supervised semantic segmentation, with only image class-labels annotations (classification accuracy 97.3%).

**Preliminaries**. The main practicality of weakly-supervised semantic segmentation methods (WSSS) is to avoid the resource-demanding manual labeling of each pixel of the categories of interest in an image, which is an impossible task for large canopies. Indeed, WSSS transforms the semantic segmentation task into the much less demanding effort of image-level class annotations. The problem is ill-conditioned and difficult, and a large literature is dedicated to the solution of it starting from Zeiler and Fergus (2014) and Zhou et al. (2016), up to most recent contributions (Chang et al., 2020; Sun et al., 2020, 2022; Wang et al., 2020; Wu et al., 2021; Zhang et al., 2021). Semantic segmentation is critical for detecting tip-burn on large canopies due to the difficulty of both identifying it on a dense set of plants and to individually localizing each tip-burned plant within the canopy, as shown in Figure 3. To ensure both identification and localization, we develop a new method for weakly-supervised semantic segmentation for the tip-burn stress (and for the visible disease in plants disease datasets) by defining a network pipeline using attention-based splitting, classification, and graph convolution.

**Figure 4:**
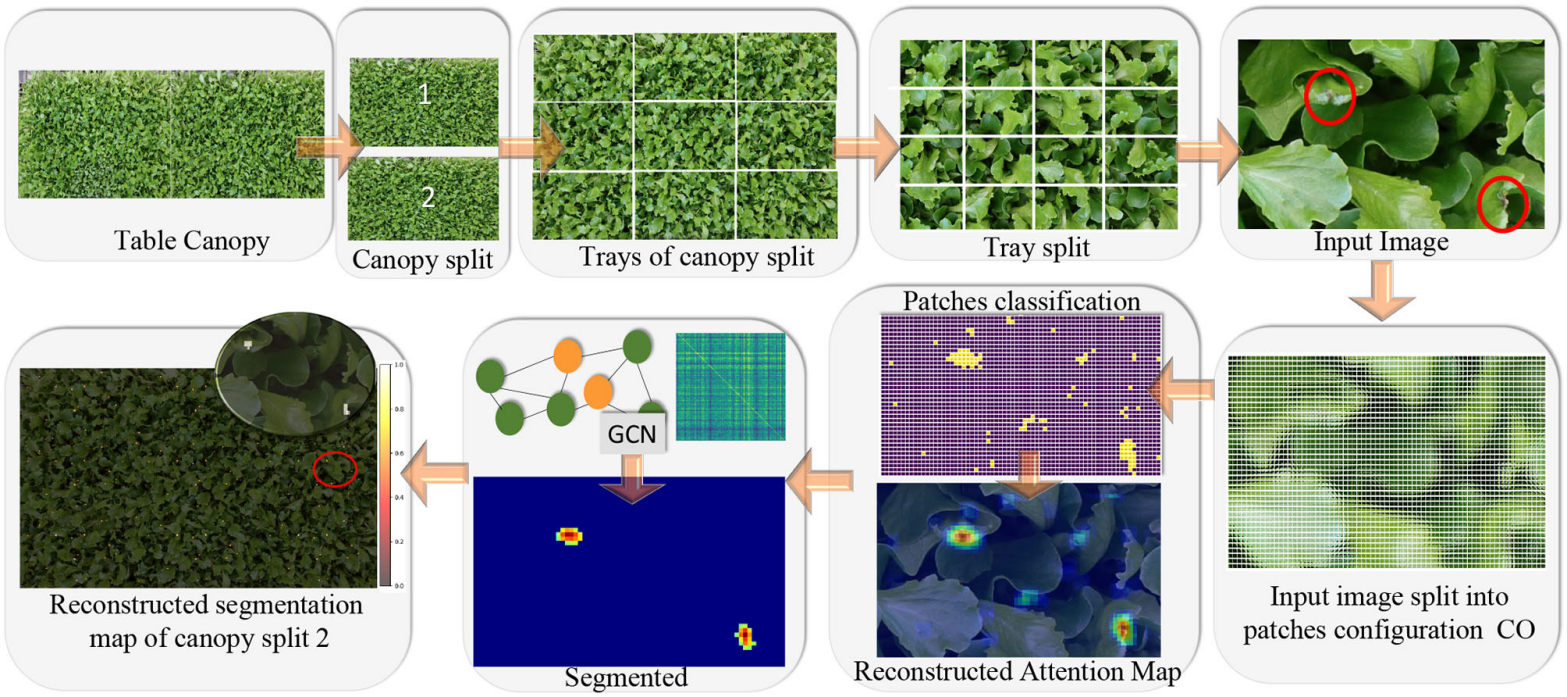
Main idea of the tip-burn semantic segmentation requiring only image-level class annotation: decompose table canopy images up to an image *X* of size 352×576×3. Split *X* into overlapping patches of size 64×64×3 and use classification trained on these patches to obtain an attention map. Use the attention map as supervision for training a convolutional graph transferring probabilities on similar patches. Finally, results are automatically merged together forming the segmentation map of the canopy.

A crucial aspect of our model is that we adopt the same classifier for both the image and the patches, suitably resized. For this idea to work, it is required that feature properties are shared between the image of the object as a whole and the image of sub-parts of the object. For example, any subset of the image of a canopy shares similar features with the image as a whole. See Figure 4, last image of the upper strip captioned as ‘input image'. Similarly, a leaf and part of a leaf have the same feature properties. This often occurs in natural images, though it is not true, for example, for a tree, which has different features for the trunk and the crown. We define this characteristic of an object feature property as the *principle of decomposition*. In this work, we show that this principle is valid for both stress detection and segmentation on large canopies and for disease detection and segmentation for leaves (from the cited datasets), which is the domain of interest in this work.

We consider a classification model *f*_ℓ_(*C*|*X, Y*, θ), where *C* indicates the class a sample image *X* belongs to, *Y* = {1, …, *c*} is the vector of training labels, ℓ indicates the size of the images accepted by the network, and θ are the network parameters. The classification model maps each sample *X* to ${\text{p}}_{X}^{\left[C\right]}$, which is the probability vector for the class *C* given *X*, as estimated by the softmax activation.

Let *X* be an *N*×*M*×*d* tensor specifying an image, and *X*^⋆^ be any connected sub-tensor of it of size *n*×*m*×*d*, with *m* ≤ *M* and *n* ≤ *N*, where connected means that chosen row and column elements *n* and *m* from *X* are consecutive. We say that *X* enjoys the principle of decomposition if, given a deep classifier *f*_*M*×*N*_(*C*|*X, Y*, θ) with probability *p* of correctly classifying *X*, with respect to classes *C*, we expect that it correctly classifies *S*(*X*^⋆^) with approximately the same *p*. Here, *S* is a suitable scaling transformation, including appropriate filtering, transforming X^*^ to *X*^⋆^′ having the same size as *X*.

**Pre-processing and classification model for tip-burn on large canopies**. Tip-burn pre-processing exposes three components. The first component is splitting the canopy image into two images of size (4.64E+ 3×6.96E + 3×3) representing half-table, then into all the sub-trays, and further each tray into 16 input images of size 352×576×3. The second component is the augmentation of the training images of size 64×64×3, by random rotation between 0 up to 90 degrees, flipping up and down and left to right, color quantization to 8 colors, zooming in by scaling and cropping, zooming out by padding, and finally by Gaussian blurring with random variance σ ∈ (0.5, 2). For the classification model, we have used as backbone Resnet50V2, trained on ImageNet 1000 and fine tuned with Global Average Pooling, drop-out (to introduce stochasticity in the training) and dense layers.

**Pre-processing and classification model for the single leaf image datasets**. For classifying the single leaf images of datasets, like PlantVillage, CitrusLeaves, and PlantLeaves, we used the same backbone as for the tip-burn. On the other hand, for weakly supervised semantic segmentation, we have also used a multi layer perceptron (MLP) to separate the background from the foreground. Profiting from the simple arrangement of a single leaf on a background of these datasets, we have automatically sampled from each image a patch of size 8×8×3 from each corner, labeling them background, and 6 patches of the same size from the image center as foreground and gave these data to the MLP to learn to separate the background from the foreground.

**Local attention by splitting with hard strides**. The main interest of splitting an image into patches with hard strides is to obtain the attention map, in a way similar to how the human gaze glimpses a scene focusing on interesting regions. Here, by hard strides, we intend strides that allow for a significant overlapping of the patches or, more specifically, strides that have a dimension much lower than the patch size.

Most of the work for attention estimation is done by the overlapping induced by the strides like when the gaze goes back to an interesting region of the scene several times. Yet, this kind of attention is *local*, as it does not capture the whole context. To obtain the context, we shall refine this spitting-based attention with spectral graph convolution, described in the next paragraph.

**The splitting process and patch classification**. Breaking an image into patches is a well-known technique (see Nowak et al., 2006; Zhou et al., 2009; Dong et al., 2011), requiring only algebraic manipulations of tensors. Consider the image *X* of size *M*×*N*×*d*. The splitting operation, along the spatial dimension, extracts from *X* patches of dimension (*p*_*x*_, *p*_*y*_, *d*). Here, the splitting combined with strides allows for overlapping the neighbors' patches according to the stride values (*s*_*x*_, *s*_*y*_). In some sense, it is like taking the inner product of *X* with the lower and upper shift matrices *A*, *A*′, and their transposed *A*^⊤^ and *A*′^⊤^ with suitable shifts, and then cropping the non-zero values. Or, similarly, convolving *X* with a shifting kernel and cropping the non zero elements. The number of obtained patches and their configuration depends on the sizes *M, N* of the image, the number of channels *d*, the patch sizes (*p*_*x*_, *p*_*y*_, *d*), and the spatial stride (*s*_*x*_, *s*_*y*_). *k*_1_ and *k*_2_ are obtained like in the convolution output, though here we do not consider padding:


(1) $$\begin{array}{l} k_{1}=\left\lceil \left(M-p_{x}\right)/s_{x}\right\rceil +1\text{ and }k_{2}=\left\lceil \left(N-p_{y}\right)/s_{y}\right\rceil +1 \end{array}$$

**Figure 5:**
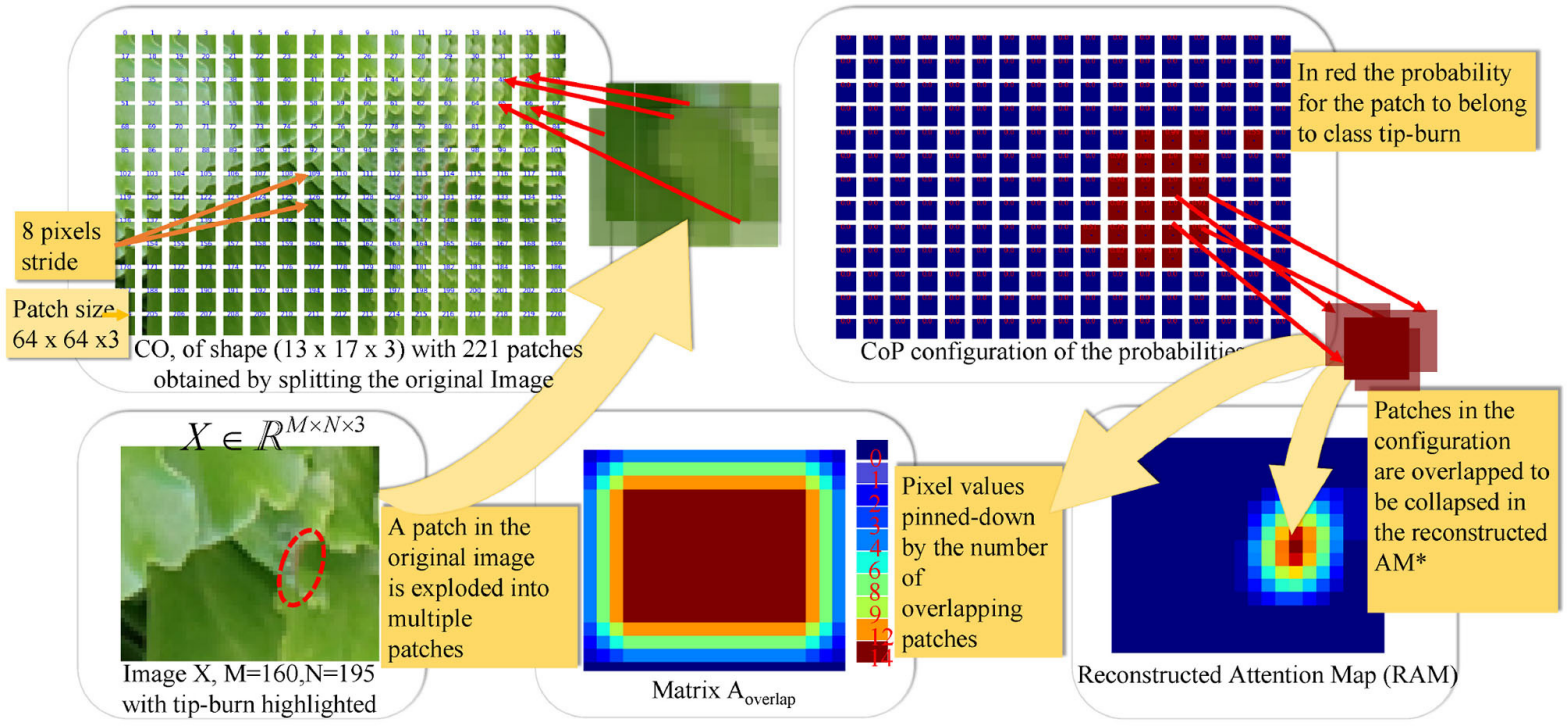
The figure illustrates the splitting process and patch classification taking as an example an image *X*∈ℝ^*M*×*N*×3^, with *M*=160 and *N*=195. The first plate above shows the *configuration* CO obtained by splitting *X* into patches of size *p*_*x*_×*p*_*y*_×*d* and a stride (*s*_*x*_, *s*_*y*_) with *p*_*x*_=*p*_*y*_=64, *d*=3 and *s*_*x*_=*s*_*y*_=8. In the example, CO has a configuration of *k*_1_×*k*_2_ patches, with *k*_1_=13 and *k*_2_=17. The plate on the upper-right shows the configuration of probabilities CoP obtained *via* the softmax by classifying each patch in CO. CoP has the same shape as CO, by fine-tuning Resnet50V2. The plates below, on the right of the image *X*, show the matrix $A_{overlap}\in {\mathbb{R}}^{M\times N}$, collecting the number of times patches overlap on a pixel when collapsing the configuration into the reconstructed image, according to the stride. The Reconstructed Attention Map (RAM) is obtained by collapsing CoP, and it has the same size as *X*.

We denote CO as a configuration of *k*_1_×*k*_2_ patches, each of size *p*_*x*_×*p*_*y*_×*d*. Namely, it is the shaping of *k*_1_ patches on a row and *k*_2_ patches on a column, shown in Figure 5.

Given *X*∈ℝ^*M*×*N*×*d*^, the configuration *CO*, and a patch *X*_*j*_ in *CO*, with 0≤*j*≤*k*_1_·*k*_2_, the patch *X*_*j*_ is resized as *S*_ℓ_(*X*_*j*_) (including required filtering modes), where ℓ indicates the size of input images accepted by the network *f*_ℓ_(*C*|*X, Y*, θ). The value of ℓ changes according to the considered dataset. For the plants, disease datasets, the classification entry corresponds to the size of the image in the dataset, may be reduced as for PlantLeaves, while a patch is proportional to the image size. This shows the extreme flexibility of the splitting process followed by classification which is adaptable to several kinds of backbones.

For each patch in the configuration CO, obtained by splitting the original image, the probability that it belongs to the class of interest (e.g., tip-burn) is estimated by the network *f*_ℓ_ resizing the patch to the input size ℓ accepted by the classification network *f*_ℓ_.

The estimation amounts to the softmax applied to the logits of the classifier *f*_ℓ_(*C*|*X, Y*, θ), here we used Resnet50V2 as a backbone. After each patch, probability to belong to *C* is estimated, a configuration of probabilities (CoP) is obtained, as shown in Figure 5. CoP has the same configuration as CO, though each patch π is defined by repeating at each pixel, the probability *p* computed by the softmax on classifying the patch. When we indicate the probability *p*_*r,c*_ of the patch located at indexes (*r, c*), we mean the probability *p*.

A mapping *h* from CoP to the reconstructed attention map (RAM) is defined by collapsing the patches in CoP into the corresponding pixels of the matrix RAM. Note that while the whole size of CoP is *k*_1_·*p*_*x*_×*k*_2_·*p*_*y*_, namely (*M*·⌈*p*_*x*_/*s*_*x*_⌉)×(*N*·⌈*p*_*y*_/*s*_*y*_⌉), with *s*_*x*_ ≪ *p*_*x*_ and *s*_*y*_ ≪ *p*_*y*_, RAM has the same spatial dimension as the original image, namely *M*×*N*. Given a patch π_*r,c*_ in row *r* and column *c* in CoP, and a pixel at location (*i, j*) in π_*r,c*_, the tuple ((*r, i*), (*c, j*)) is mapped by *h* to the pixel (*x, y*) in RAM, as follows:


(2) $$\begin{array}{l} {\left(x,y\right)}_{RAM}=\left(h\left(r,i\right),h\left(c,j\right)\right)=\left(\left(i+r\cdot s_{x}\right),\left(j+c\cdot s_{y}\right)\right)\\ \text{for }\pi_{r,c}\in CoP\text{ and }\left(i,j\right)\in \pi_{r,c}. \end{array}$$

Given Equation (2), we also obtain a matrix *A*_*overlap*_ by counting all times a pixel from CoP hits the corresponding pixel of RAM. Indeed, this matrix specifies how many overlapping patches contribute to a pixel in RAM:


(3) $$\begin{array}{l} A_{overlap}{\left(\left(h\left(r,i\right),h\left(c,j\right)\right)\right)}^{\left(t\right)}=A_{overlap}{\left(\left(h\left(r,i\right),h\left(c,j\right)\right)\right)}^{\left(t-1\right)}+1. \end{array}$$

The matrix *A*_*overlap*_ is used to count the accuracy of the classification at the pixel level, and it allows to suitably average RAM. The averaged RAM is obtained as follows:


(4) $$\begin{array}{l} RAM^{⋆}=RAM/A_{overlap} \end{array}$$

Figure 5 shows an example of CO representation, of CoP, of the matrix *A*_*overlap*_, of RAM given a random image *X* with tip-burn highlighted. Where, here and from now on, we denote *RAM*^⋆^ RAM.

We can see that the accuracy of RAM is determined by the strides. For example, if the stride is *s*_*x*_ = *s*_*y*_ = 10, we have an accuracy at the level of a region of size 10×10, and if the stride is *s*_*x*_ = *s*_*y*_ = 1, the accuracy is at the pixel level allowing to effectively label each pixel. Clearly reducing the stride increases the number of patches of the same image. The average increase of the number of pixel is by a factor of 9.

**Refining by graph convolution**. The RAM results in pseudo segmentation masks for the tip-burn stressed leaves found in the RGB image, following the pipeline splitting-classification-reconstruction. Differently from CAM (Zeiler and Fergus, 2014; Zhou et al., 2016), RAM highlights in the same map all objects of interest quite accurately. Moreover, while in CAM the result is obtained by the gradient of the softmax outcome, with respect to the last feature map, which has very low resolution, thus requiring significant resizing inducing blurring, here we do not need any resizing, as we can obtain the original image by a single step merging, according to Equation 2. Despite classification accuracy for tip-burn is 98.3%, and for the other datasets is no less than 96%, there is still noise on the attention map because classification is done on *S*_ℓ_(*X*), namely on the resized patch, given the decomposition principle.

**Figure 6:**
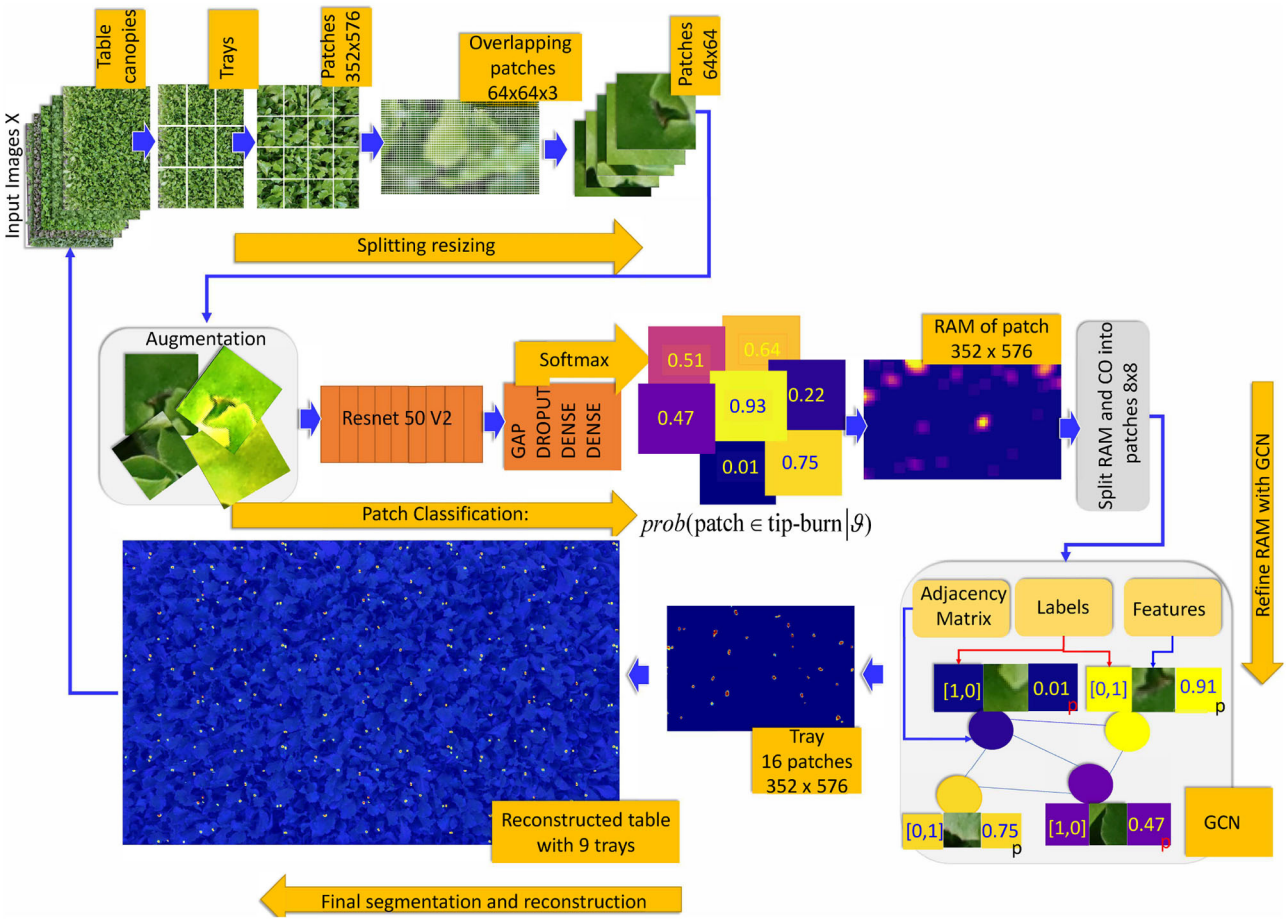
Overview of the weakly supervised semantic segmentation process of tip-burn leaves stress supervised with image-level class annotation only. Above, the splitting process starts at images *X* ∈ ℝ^*M*×*N*×3^, *M*=4, 640, *N*=6, 960. Note that starting from patches of size 352×576×3 the splitting process plays with overlapping patches of size 64×64×3 with a hard stride of 8 pixels. The classification is trained to detect tip-burn stresses, and *via* the softmax to predict a probability for each patch to belong to the class tip-burn. After classification, a reconstruction step obtains the Reconstructed Attention Map (RAM) for patches of size 352×576×3. These are again split into patches 8×8×3 and used to supervise the Graph Convolutional network (GCN). Namely, the GCN features nodes are the flattened 8×8×3 patches and the labels are one hot encoded vectors obtained from the classification predictions, see the plate with the GCN, The GCN estimates a refined semantic segmentation of pixels. A final reconstruction does the inverse splitting process reconstructing the table canopies from patches.

Comparing the size of a patch in CoP and the size of the probabilities highlighted in RAM in Figure 5, we can note they have different sizes. This is due to overlapping and projection, which augment the resolution of the probability from uniform in a patch of size 64×64 to uniform on a patch of size 8×8. Indeed, the RAM probability resolution is 8×8. Having in RAM a higher probability resolution, we re-propose the splitting into sub-patches with size (*s*_*x*_, *s*_*y*_, *d*), namely of size 8×8×*d*, *d* ∈ {1, 3}, for both the RGB image of size 352×576×3 (see the paragraph above, on Preprocessing and Classification, and Figure 6) and the RAM of size 352×576×1. A schema of this further splitting follows:


(5) $$\begin{array}{r} CO_{new}\leftarrow \text{size}=42\times 72\times 3,\text{sub-patch size}=8\times 8\times 3,\\ \text{num of sub-patches}=3168\\ CoP_{new}\leftarrow \text{size}=42\times 72\times 1,\text{sub-patch size}=8\times 8\times 1,\\ \text{num of sub-patches}=3168 \end{array}$$

The goal is to use CoP_*new*_ as supervision for training a convolutional graph network (GCN) improving the semantic segmentation accuracy obtained by classifying the patches. This further splitting step obtains a *CO*_*new*_ and a *CoP*_*new*_, as specified in Equation (5), from which we obtain the features and the labels for the GCN. The softmax of the GCN classifies the nodes of the GCN, inducing an effective semantic segmentation for tip-burn for each image in the dataset, and similarly for the other datasets.

Following Kipf and Welling (2016), a number of approaches have experimented with graph convolution (GCN), especially on non grid structures. Though, recently, an increasing interest is devolved to apply graph convolution on images, for segmentation and attention purposes, such as Li and Gupta (2018) and Hu et al. (2020). Here, we apply an unsupervised node classification, conditioning the graph model both on the data and on the adjacency matrix *via* graph convolution. As a matter of fact, we are going to generate the adjacency matrix for the graph G = (V, E), fully unsupervised. We construct a graph for each RGB input image *X* of size 352×576×3, fixing the size of the nodes, so as to put the graphs in a batch.

We take the patches as node features, labeling them with a one hot encoding vector obtained by thresholding the score *p*_*c,r*_ in *CoP*_*new*_ of patch π_*c,r*_ ∈ *CO*_*new*_. More precisely, we flatten each mini-patch of size 8×8×3 into a vector **x** ∈ ℝ^*k*^, *k* = 192 and stack all the flattened patches into a matrix $X_{\varphi }\in {\mathbb{R}}^{n\times k}$, *n* = 3168. To obtain a corresponding ordering, we use an index function *idx*:(*r, c*) → *i*, *idx*(*r, c*)=*w*(*c*−1)+*r*=*i* with *w* the number of rows, *r* and *c* the row and column indexes in *CO*_*new*_ and in *CoP*_*new*_, respectively, and *i* the corresponding index in *X*_φ_. *X*_φ_ is the input matrix to the network. At each layer of the network, a feature matrix is generated, starting with *X*_φ_.

To connect subsets of nodes, based on their feature similarity, we generate the adjacency matrix *Adj*, which is symmetric and of size *n*×*n*, as follows. We keep the indexing *idx* to maintain the correspondence between *CO*_*new*_ and *X*_φ_ and between *CoP*_*new*_ and the labels. For each sub-patch, we estimate a non-parametric probability by computing the histogram using both the RGB and the HSV color transformation of the sub-patch and collapsing the 64·3·2 vector into a histogram with 64 bin-edges. For each pair of histograms *q*_*i*_, *q*_*j*_, we compute the Shannon-Jensen divergence:


(6) $$\begin{array}{l} \text{JSD}\left(q_{i}\|q_{j}\right)=0.5\text{KL}\left(q_{i}\|m\right)+0.5\text{KL}\left(q_{j}\|m\right)\\ \text{with}\\ m=0.5\left(q_{i}+q_{j}\right)\text{ and KL}\left(q_{i}\|m\right)=\sum_{x\in X}q_{i}\left(x\right)\log\left(q_{i}\left(x\right)/m\left(x\right)\right) \end{array}$$

The choice of JSD is required by the need of the adjacency matrix *Adj* to be symmetric. Then, two nodes *v*_*i*_, *v*_*j*_ feature vectors **x**_*i*_ and **x**_*j*_ are similar, hence connected by an edge *e*_*i,j*_∈E if *JSD*<β, we have chosen β=0.005 for the tip-burn dataset. Given the *n* nodes in V, the diagonal degree matrix *D* adds for each node the number of its connected ones. The normalized graph Laplacian matrix is $L_{norm}=I_{n}-D^{-\frac{1}{2}}Adj\;D^{-\frac{1}{2}}=U\Lambda U^{⊤}$. Here, Λ is the matrix of the eigenvalues and *U* is the orthogonal eigenvectors. The graph convolution *g*_θ_(*L*_*norm*_) ⋆ *X*_*j*_ using *L*_*norm*_ is the spectral convolution, based on obtaining parameter filters from the eigenvectors of *L*_*norm*_ in the Fourier domain. Several simplifications have been proposed, we refer the reader to Defferrard et al. (2016) for spectral convolution in the Fourier domain and the approximation of the *L* eigenvectors by Chebyshev polynomial up to the K-th order. Kipf and Welling (2016) obtain a GCN by a first-order approximation spectral graph convolution. They define *K* = 1 and reduce the Chebyshev coefficients to a matrix of filter parameters.

The feed forward propagation of the GCN is recursively defined as:


(7) $$\begin{array}{l} H^{\left(t+1\right)}=\sigma \left(ÂH^{\left(t\right)}W^{\left(t\right)}\right),\text{ with }H^{\left(0\right)}=X_{\varphi } \end{array}$$

Here σ is an activation function, $H^{\left(t\right)}=\left\{{\text{h}}_{1}^{\left(t\right)},\ldots ,{\text{h}}_{n}^{\left(t\right)}\right\}$ are the hidden vectors of the *t*-th layer, with ${\text{h}}_{i}^{\left(t\right)}$ the hidden feature vector of the node *v*_*i*_. Â is defined as follows. *A* = *Adj* + *I*_*n*_, to include self loops, since nodes are self similar, ${\tilde{D}}_{ii}=\underset{j}{\sum }A_{ij}$ and $Â={\tilde{D}}^{-\frac{1}{2}}A{\tilde{D}}^{-\frac{1}{2}}$, so as to be normalized, *I*_*n*_ is the identity matrix of size *n*×*n*. The role of Â is to aggregate information from connected nodes. *W*^(*t*)^ is the weight matrix to be learned. The dimensions are as follows:


(8) $$\begin{array}{l} Â\in {\mathbb{R}}^{n\times n},H^{\left(t\right)}\in {\mathbb{R}}^{n\times u_{t}},W^{\left(t\right)}\in {\mathbb{R}}^{u_{t}\times u_{t+1}} \end{array}$$

Here, *u*_*t*_ and *u*_*t*+1_ are the sizes of the hidden layers. A 3 layer GCN has the form:


(9) $$\begin{array}{l} Z=g\left(X_{\varphi }|Â,W\right)=\text{softmax}\left(G\right) \end{array}$$

Where the softmax is applied row-wise and


(10) $$\begin{array}{l} G=Â\text{ ReLU}\left(Â\left(\text{ReLU}\left(ÂH^{\left(0\right)}W^{\left(0\right)}\right)W^{\left(1\right)}\right)W^{\left(2\right)}\right) \end{array}$$

The optimization of the GCN uses cross-entropy loss on all labeled nodes (Kipf and Welling, 2016), where here the labels are the one hot encoded values obtained from *RAM*_*new*_. Let us denote by I the indexes of the nodes and by *Y*_*i,l*_ an indicator which has value 1 if node *v*_*i*_ has label *l*:


(11) $$\begin{array}{l} L=-\underset{i\in I}{\sum }\overset{n}{\underset{l=1}{\sum }}Y_{i,l}\log Z_{i,l} \end{array}$$

According to the number *K* of layers, a GCN convolves the *K*-hop neighbors of a node, essentially clustering similar nodes, according to their probability labels and features. We use simple 3 layers GCN, since in the end tip-burn stresses on leaves are very small and rare. An overview of the whole learning process is given in Figure 6.

The GCN adjusts the RAM by looking at the context, going beyond the localized estimation of splitting plus classification. GCN estimates the probability that a node, corresponding to features of a patch 8×8×3, belongs to tip-burn or not, by updating the belief that two patches are similar. At the end of the training, *CoP*_*new*_ is updated with the new distribution. In Table 2, in Section 5, we show the advantages of the GCN by ablation.

**Reconstruction**. Given the initial image of the dense canopy, the question to be explored is “which plant suffers tip-burn stress and where it is?” including counting would not be so useful. Consider that when tables are unrolled, from the position of the plant on the table, it is possible to go back to the cell the plant comes from, and possibly revise its growing conditions, or make useful statistics. It is therefore pivotal to localize the stress segment on the table image. It turns out that by the proposed model it is extremely easy.

In fact, as noted in the paragraph on splitting, reconstruction is automatically done by projecting back a pixel in CoP into a pixel in RAM by Equation (2). Obviously, it can be done for any image, not only for the maps but also for RGB images.

Reconstruction is done both when the stride *s*_*x*_>1 and *s*_*y*_>1, that is when the splitting generates sub-images that overlap and, obviously, when they do not overlap. This, in fact, can be done for all the steps of splitting, from the table canopy up to *Ram*_*new*_ and for its dual RGB image, and again back to the large table canopy.

The back process requires preserving just the patches size for the maps and the scores, at each layer of the splitting. Then, the process is simply recursively applied to go from the patch up to the image of the whole canopy. Note that for the semantic segmentation, we need only to preserve the score vectors estimated by GCN. An image of a partial reconstruction of the half table is given in Figures 4, 6.

## 4. Application of the Model to Other Datasets

As gathered in the introduction, we have collected three datasets, namely PlantLeaves (Chouhan et al., 2019), PlantsVillage (Hughes and Salathe, 2016), and Citrus leaves (Rauf et al., 2019), to evaluate our approach. Usually these datasets are tested for classification, which has nowadays obtained striking results. Here, instead, we consider the semantic segmentation of the leaves lesions using only images class-labels, which is actually the only information available for these datasets.

Our goal here is to discuss mildly classification and most of all the whole pipeline we used to segment both the leaves and the disease spots and lesions. Clearly, segmenting the disease is more difficult when the leaf is almost completely covered by the disease spots, which are discolored regions or dark necrotic spots. As we shall see, the best results are actually obtained for CitrusLeaves and PlantLeaves, where the disease spots are localized.

From each dataset, we have chosen a class of diseases and the corresponding healthy images, for segmentation. For PlantVillage, we used the whole dataset, but we have chosen only Pepper Bell bacterial and Pepper Bell healthy for segmentation. Consider that the only burden for classification once the model is defined is to load the data. In turn, the model is just a fine-tuning of an already existing model, such as Resnet50V2. All parameters and accuracies for each network model available for fine-tuning classification are provided on the Keras Application page.

PlantLeaves consists of 4,502 images of healthy and diseased leaves divided into 22 categories including species and disease. From this dataset, we have chosen Pomegranate (P9) both diseased and healthy. There are 272 images of diseased Pomegranate (P9) and 287 healthy ones.

PlantVillage consists of 54,303 images of healthy and diseased leaves divided into 38 categories including species and disease. It is possible to download either the augmented or the non augmented set of images. As gathered above we have considered the whole dataset for classification, PepperBell healthy and PepperBell Bacterial spot for segmentation. PepperBell Bacterial spot are 998 images, and PepperBell healthy are 1,478 images.

CitrusLeaves consists of 594 images of healthy and diseased leaves with 4 diseased categories and one healthy. We have chosen healthy and Canker. Canker contains 163 images, while healthy contains 58 images.

**Figure 7:**
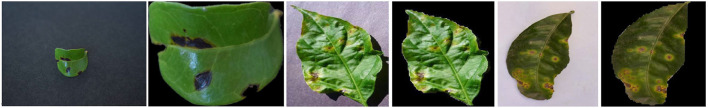
Preliminary leaves segmentation for PlantLeaves, PlantVillage, and CitrusLeaves, from left to right in the order.

The model for the above indicated datasets, from splitting up to segmentation, is similar to the tip-burn stress segmentation, starting from splitting. Yet, the preprocessing is quite different. Preprocessing, for the three datasets consists of removing the background and tightening the image within its bounding box. This last step is crucial for complying with the principle of decomposition, discussed at the beginning of the method, and also for avoiding overfitting due to background. We have automatically sampled from each image, the four corners with 8×8×3 pixels and 6 patches of the same size from the image center. We have then defined a MLP that could accept sub-patches of the size 8×8×3 to separate background and foreground. Some results, compared with the original image are shown in Figure 7. Note that the background has a value (0, 0, 0), thus not influencing the CNN classification.

Being the image of PlantLeaves of size 4E + 3×6E + 3, we reduce them to 264×400 after automatic cropping with the MLP. On the other hand, we resize both CitrusLeaves and PlantLeaves to their original size 256×256, after automatic cropping with MLP. We do augmentation by flipping up and down, left and right, and blurring with a Gaussian filter with random variance σ ∈ (0.5, 2), for both CitrusLeaves and PlantLeaves. We have not augmented PlantVillage, since it comes already augmented.

Also, differently from the model for tip-burn stress detection, for these datasets, we do the first splitting to a size of 70×70, with the same stride of *s*_*x*_ = *s*_*y*_ = 8, for tip-burn, and then we resize each patch to the original image size for classification. For classification, we have fine-tuned Resnet50V2, as for tip-burn data. The remaining of the model, from further splitting up to the GCN and the reconstruction, here just for the leaves, follows the same steps, which are the relevant novelties.

## 5. Experiments and Results

### 5.1. Setup

The whole model is implemented in Tensorflow 2.5, on a GeForce RTX 3080, 300 HZ. For the ResNet50V2, we use the Keras API in Tensorflow. We used the Keras functional API for fine tuning the model, with all the provided advances, such as early stopping, and learning rate decay. For early stopping we used *patience* 4, with delta 0.001. For reducing the initial learning rate when on a plateau, we used a factor of 0.2 and a minimum learning rate of 0.001 starting with an initial learning rate of 0.1. For the loss, we used categorical cross entropy with Adam as the optimizer (Kingma and Ba, 2014).

For the splitting and reconstruction, we use Tensorflow GradientTape, as the gradient computes both *A*_*overlap*_ and the mapping between *CoP* and *RAM* for both Equations (2) and (3).

We have implemented a good part of the GCN including the adjacency matrix, the features vectors and the joining step, to transfer probabilities, in Tensorflow. We used much intuition from DGL, an open-source graph library introduced by Zheng et al. (2021), though DGL is implemented in PyTorch. We also get inspired by Spektral of Grattarola and Alippi (2021), an open-source Python library, for building graph neural networks with TensorFlow and Keras interface. As specified in the Method Section, we have been using cross entropy loss and Adam optimizer like in the classifier together with early stopping.

### 5.2. Comparison With State of the Art

The main contribution of our work is weakly-supervised semantic segmentation with the only supervision being the image class labels, whether there is tip-burn or not. For classification, we have been using Resnet50V2, because it is quite flexible, and fine-tuned it. As we have already mentioned, we expect that if *f* is a classifier that classifies correctly *X* with probability *p*, if the classifier generalizes well, it would classify the resized *X*, namely *S*(*X*), approximately with the same probability *p*. This is shown to be correct for tip-burn and plant disease datasets CitrusLeaves, PlantLeaves, and PlantVillage, according to the principle of decomposition.

**Stress and disease detection**. For training tip-burn CNN classification, we used 30 out of 43 images and compared our work with Gozzovelli et al. (2021), where DarkNet was used. For classification of the plant disease datasets, we considered the following recent works: Sujatha et al. (2021), Khattak et al. (2021), and Syed-Ab-Rahman et al. (2022) for CitrusLeaves; Mohameth et al. (2020), Mohanty et al. (2016), Chen et al. (2020), Agarwal et al. (2020), and Abbas et al. (2021) for PlantVillage; for PlantLeaves, we expose only our approach as there are no recent contributions.

**Figure 8:**
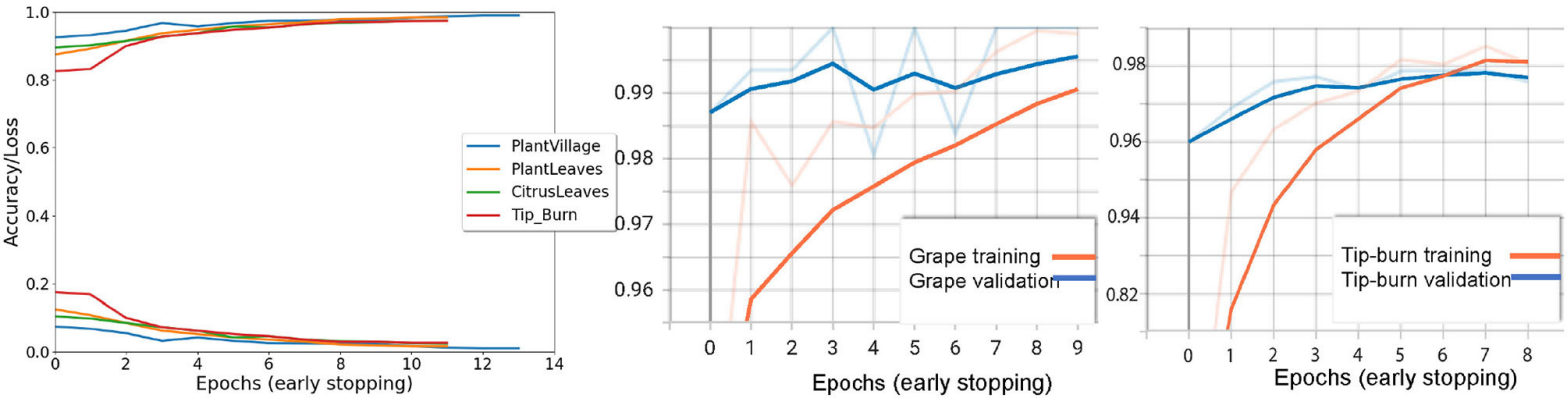
The first graph on the left shows the validation accuracy and the loss for Tip-burn, PlantsVillage, CitrusLeaves, and PlantLeaves, with patience 4 for both early stopping and for updating the learning rule. We can observe that the maximum number of epochs is 13 for PlantsVillage. In the central graph, we see a paradox, validation hits 1% before training, for the grape class in PlantsVillage. On the right, we observe the convergence for tip-burn at 6 epochs.

Results are shown in Table 1, considering validation accuracy, as usual. The best accuracy in class is highlighted in bold. We note that for a number of species in PlantVillage, we obtained a validation accuracy of 1.0, in few epochs. Since we use early dropping, this was not caused by overfitting, as shown in Figure 8, where it can be observed that for all datasets we have a small number of epochs. Note that we have also removed the background because, according to Barbedo (2019b), accuracy drops without the background. It seems possible that the background induces overfitting. In Figure 8, we show some results motivated by changing the patience value in early stopping.

**Table 1:** Tip-burn stress and plant disease detection.

**References**	**Method**	**Tip-Burn**		**CitrusLeaves**				**PlantVillage**		**PlantLeaves**
		**Acc**	**F1**	**Acc**	**Acc**	**F1**	**F1**	**Acc**	**F1**	**Acc**
				**Canker**	**Healthy**	**Canker**	**Healthy**	**Whole**	**Pommg**.	
Our approach	Resnet50V2 (fine t.)	**0.978**	0.983	**0.964**	**0.975**	0.981	0.963	0.989	0.958	**0.984**
Gozzovelli et al., 2021	DarkNet	0.961	0.960							
Sujatha et al., 2021	InceptionV3/			0.937	0.965					
	VGG16									
Khattak et al., 2021	Own method			0.945						
Syed-Ab-Rahman et al., 2022	Faster R-CNN			0.945						
Mohanty et al., 2016	AlexNet							**0.993**	0.972	
	GoogleLeNet									
Chen et al., 2020	Own Method							0.918		
Mohameth et al., 2020	Resnet50									
	InceptionV3									
	MobileNet									
Agarwal et al., 2020	VGG16							0.912		
Abbas et al., 2021	DensNet121 +							0.971	0.97	
	Synthetic Images									
Sharma et al., 2020	Own method							0.986		

*Best results are highlighted in bold*.

We split the leaves datasets 70/30% between train and validation plus test as in Mohanty et al. (2016). As shown by Mohanty et al. (2016), GoogleLeNet is the best backbone for plant disease detection, and we recall that the assessed performance of Resnet50V2 on the Imagenet validation set is 0.760 on Top-1 accuracy and 0.930 on Top-5 accuracy.

### 5.3. Tip-Burn Segmentation and Plant Datasets Segmentation

**Testing accuracy by manually labeling ground-truth**. Typically, accuracy metrics for segmentation are *F*_1_, in the context of segmentation referred to as Dice similarity coefficient, and Intersection over Union (IoU), both required to compute the corresponding pixels (true positive), the exceeding pixels (false positives) or lacking pixels (false negative) between the ground truth mask and the estimated masks. Because, in none of the available datasets, we have ground truth masks available, we introduce the patch-based method that is not too demanding to obtain an approximate Dice coefficient. Here, approximate means that instead of computing the pixels we compute the super-pixels and also it means that we use a reduced number of test samples.

We consider 1 test image from the table canopy images and 20 test images for each of the plant disease datasets. Note that 1 test image is the half image of a table canopy, and it amounts to 346,464 images of size 64×64×3, that is 2, 406·16·9.

Now, assuming that we have segmented the test images, by the automatic decomposition and recomposition process, by definition of the model, we have made available all the patches that contribute to the final estimated segmentation. These patches are actually vectors *Z* holding the probability that the corresponding RGB vector is of class tip-burn or not, as estimated by the GCN and similarly, for the other datasets. At the same time, according to the described model, there is a one to one correspondence between the patches in *CoP*_*new*_ and *CO*_*new*_ and there is a correspondence, by Equation (2), between the patches and the attention map *RAM*, hence the image, and the final segmentation map estimated. So, it is enough to choose the patches in *CO*_*new*_. The manually chosen patches are immediately aligned with *CoP*_*new*_ and the final segmentation. That is, suppose we have chosen a patch *X*_*j*_ which will be of size 8×8×3, by definition of the model, then we have automatically selected 64 pixels, and the process is significantly sped up. Once the patches are selected, we know the corresponding value in the segmentation map and can compute both the Dice similarity coefficient at sub-patch or super-pixel level, instead of pixel level, and the IoU. Let *X* = {*X*_*j*_|*X*_*j*_ ∈ selected}, where each selected patch has value 1, and *Y* the corresponding patches in $COP_{new}^{\prime }$ with value *Z* computed by the GCN, the *DSC*_*patch*_= 2(*X*∩*Y*)/(|*X*| + |*Y*|), with | · | the cardinality.

Table 2 gives the results for the approximate meanIoU and F1 (Dice similarity coefficient), computed according to sub-patches (super-pixels) in place of pixels, and according to a limited subset of the test images. We give some ablation too.

**Table 2:** Segmentation and ablations.

**Similarity metrics**	**Tip-burn**	**Citrus leaves**	**PlantVillage**	**PlantLeaves**
**Segmentation by thresholding the Attention Map RAM**				
Dice similarity coefficient (DSC/*F*_1_)	0.7827	0.6996	0.6799	0.7326
Intersection over union (IOU)	0.6430	0.5380	0.5150	0.5780
**2 Layers GCN**				
Dice similarity coefficient (DSC/*F*_1_)	0.8386	0.7277	0.6868	0.7908
Intersection over union (IOU)	0.7220	0.5720	0.5230	0.6540
**3 Layers GCN**				
Dice similarity coefficient (DSC/*F*_1_)	0.8499	0.7326	0.6292	0.7974
Intersection over union (IOU)	0.7390	0.5780	0.4590	0.6630
**Doubling the number of nodes in the graph**				
Dice similarity coefficient (DSC/*F*_1_)	0.7797	0.7105	0.6217	0.7021
Intersection over union (IOU)	0.6540	0.551	0.4511	0.541

**Ablation**. Consider first the segmentation using just thresholding of the attention map RAM. Introducing the CGN with two layers, we observe an improvement for all models. Extending the GCN to three layers, we observe that accuracy improves for all models but for PlantVillage. It is interesting to note also that doubling the number of nodes in the GCN lowers the accuracy for all models, which is reasonable because we have to choose patches with lower probability of being tip-burn.

**Figure 9:**
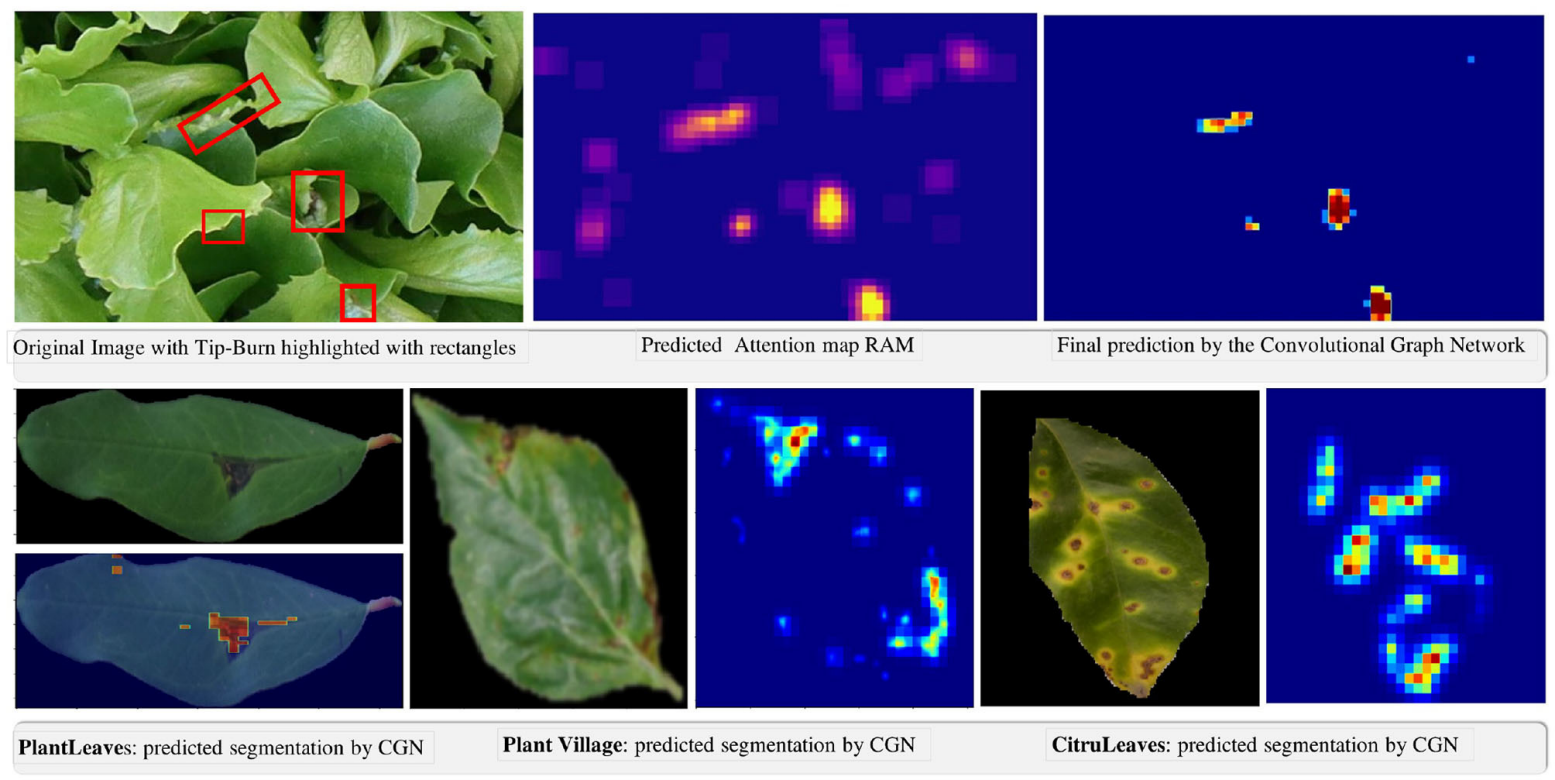
Qualitative results of the weakly supervised semantic segmentation of tip-burn stress and of disease spot and lesions on PlantVillage, PlantLeaves, and CitrusLeaves.

In Figure 9, we provide some qualitative results facilitating an understanding of the extremely good results of the model.

## 6. Conclusion

In this paper, we have introduced a new method for detection and localization of tip-burn stress in large plant canopies grown in plant factories. The idea is very simple to implement, and the only supervised step is a classification of the image, namely just knowing the class in the image. We have shown that the method obtains quite nice refined weakly self-supervised segmentation for tip-burn stress.

We have tested our method both on publicly available datasets, such as PlantVillage, PlantLeaves, and CitrusLeaves, and in operating conditions inside a plant factory showing the flexibility of our model. The results show that plant stress detection and localization can be done automatically in Controlled Environment Agriculture conditions.

## Data Availability Statement

The raw data supporting the conclusions of this article will be made available by the authors, without undue reservation.

## Ethics Statement

Written informed consent was obtained from the individual(s) for the publication of any potentially identifiable images or data included in this article.

## Author Contributions

All authors listed have made a substantial, direct, and intellectual contribution to the work and approved it for publication.

## Funding

The research has been partly funded by Agricola Moderna.

## Conflict of Interest

BF was employed by Agricola Moderna. The remaining author declares that the research was conducted in the absence of any commercial or financial relationships that could be construed as a potential conflict of interest.

## Publisher's Note

All claims expressed in this article are solely those of the authors and do not necessarily represent those of their affiliated organizations, or those of the publisher, the editors and the reviewers. Any product that may be evaluated in this article, or claim that may be made by its manufacturer, is not guaranteed or endorsed by the publisher.
